# Clinician Stigma and Willingness to Treat Those With Sexual Interest in Children

**DOI:** 10.5964/sotrap.5463

**Published:** 2022-03-31

**Authors:** Kailey Roche, Skye Stephens

**Affiliations:** 1 Department of Psychology, Saint Mary's University, Halifax, Canada; Centre for Criminology (Kriminologische Zentralstelle – KrimZ), Wiesbaden, Germany

**Keywords:** sexual interest in children, minor attraction, stigma, clinicians, treatment, decision making

## Abstract

The present study examined North American clinician stigma and willingness to treat those with sexual interest in children. Clinicians (N = 101) were randomly assigned to a vignette describing a referral of a client with a sexual interest in children and asked whether they would accept the client for treatment. Vignettes differed in what the client was seeking treatment for (low mood or managing sexual interest) and sexual offence history (no offence history or a contact offence against a child). Clinicians with lower stigma were more likely to accept a referral for a client with sexual interest in children and, in general, clinicians were least likely to accept a referral for a client with a sexual offence against a child looking to manage their sexual interest. Implications and future directions are discussed.

## Highlights

- Study examines clinician willingness to treat those with sexual interest in children
- Willingness to treat was associated with lower stigma scores
- Willingness to treat was associated with client’s presenting issue
- Results highlight need for clinician training to work with people with sexual interest in children

Researchers have explored mental health needs and access for those with sexual interest in children in community-based samples. Levenson and Grady (2019) found some participants desired treatment for managing their sexual interest, but reported other concerns such as depression, anxiety, and suicidal ideation. These results suggest a need for comprehensive mental health services in the population, which could have the dual aim of child sexual abuse (CSA) prevention and promotion of personal wellbeing. Although 82% of respondents in one survey agreed they would benefit from mental health services, 58% indicated they would not seek help, with many citing concerns around being stigmatized by the clinician (e.g., being judged; B4U-ACT, 2011a).

The difficulty individuals with sexual interest in children face in accessing treatment is a significant problem. In one study, 48% of participants reported that not seeking help resulted in issues like depression, low self-esteem, suicidal ideation, and isolation (B4U-ACT, 2011b). Jahnke, Schmidt, et al. (2015) posited that stigma-related stress may negatively impact emotional and social functioning, offence-supportive attitudes, and treatment motivation, which may increase risk of CSA. Given the proposed negative sequalae of not receiving treatment, the present study focuses on clinician stigma and its impact on willingness to treat people with sexual interest in children.

## Clinician Stigma and Competency

Most literature on willingness to treat people with sexual interest in children has been limited to a handful of European studies. Stiels-Glenn (2010) found 95% of their German psychotherapist sample were unwilling to treat those with sexual interest in children and a similar result was found among Swedish therapists (Niehaus et al., 2020). Low willingness to treat is present regardless of offending history; for example, in a small sample of Russian sexologists (*n* = 26), over one quarter believed those with sexual interest in children should be incarcerated, regardless of offence status (Koops et al., 2016). More promising results were reported by Jahnke, Philipp, and Hoyer (2015) who found that in a sample of psychotherapists in training, 80% were willing to treat non-offending individuals with sexual interest in children and held more positive attitudes toward this group, compared with previous studies.

Although willingness to treat those with sexual interest in children is in part impacted by pre-existing negative attitudes towards this group (Niehaus et al., 2020), it may also be impacted by clinicians’ lack of experience and knowledge of client presenting issues. For example, 20% of psychotherapists in Stiels-Glenn (2010) stated their lack of training and experience accounted for their low willingness to treat this population. Therefore, the unwillingness to treat this client population may be partially explained by competency to address various presenting issues. Indeed, several studies have found that mental health professionals have limited training in sexual health (e.g., Reissing & Giulio, 2010), which would arguably include even less training in treating atypical sexualities.

It is understandable that clinicians without competency would be hesitant to accept a referral for a client with sexual interest in children, especially if they wanted treatment to help manage this interest or had a sexual offending history. Nonetheless, many clients with sexual interest in children without an offence history express interest in receiving mental health treatment for a variety of concerns (Cantor & McPhail, 2016), which would arguably fall under the scope of practice of most clinicians. For example, a client with sexual interest in children may seek out therapy for an adjustment disorder related to the loss of a loved one. Arguably more general mental health treatment could be provided by generalist clinicians, given the expected focus of treatment.

## Present Study

We examined correlates of willingness to treat people with sexual interest in children with a focus on stigma, client presenting problem, and competency. We hypothesized that clinicians without competency to treat clients with sexual interest in children would hold a greater degree of stigma than those with competency. Lastly, we hypothesized that clinicians with a greater degree of stigmatizing attitudes would be less likely to accept a referral to treat a client with sexual interest in children, regardless of the client’s presenting issue.

## Method

### Participants

We recruited 132 North American clinicians to complete an online vignette study of willingness to treat people with sexual interest in children and to attend a workshop to increase competency to treat those with sexual interest in children. Clinicians and students in professional mental health programs were eligible if they provided mental health assessment/treatment within the past 12 months and understood English (see Table 1 for descriptive information about the sample). Clinicians were excluded if they did not complete the survey (*n* = 30) or were not from North America (*n* = 6).

**Table 1:** Participant Demographic Information (N = 101)

Variable	*M* (*SD*) or %
**Age (*n* = 101)**	40.56 (12.49)
Sex (*n* = 101)	
Female	67.3% (*n* = 68)
Male	32.7% (*n* = 33)
Not listed	–
Gender (*n* = 101)	
Woman	66.3% (*n* = 67)
Man	31.7% (*n* =32)
Transwoman	–
Transman	–
Gender fluid/gender queer	2.0% (*n* = 2)
Not listed	–
Race and Ethnicity (*n* = 100)	
Asian – East	–
Asian - South East	–
Black – African	–
Black - North American	2.0% (*n* = 2)
Indigenous/Aboriginal	1.0% (*n* = 1)
Latin American	–
Métis	–
Middle Eastern	–
White – European	13.0% (*n* = 13)
White – North American	77% (*n* = 77)
Mixed Heritage	3.0% (*n* = 3)
Not listed	4.0% (*n* = 4)
Country (*n* = 101)	
Canada	44.6% (*n* = 45)
United States	55.4% (*n* = 56)
Profession (*n* = 101)	
Physician	2.0% (*n* = 2)
Psychologist	36.6% (*n* = 37)
Counsellor/Psychotherapist	9.9% (*n* = 10)
Occupational Therapist	2.0% (*n* = 2)
Social Worker	23.8% (*n* = 24)
Mental Health Nurse	1.0% (*n* = 1)
Behavioral Therapist	2.0% (*n* = 2)
Student	18.8% (*n* = 19)
Other	4.0% (*n* = 4)
Highest Degree Obtained (*n* = 101)	
Student in professional program	19.8% (*n* = 20)
Bachelor’s Degree	4.0% (*n* = 4)
College Degree	1.0% (*n* = 1)
Medical Degree	2.0% (*n* = 2)
Master’s Degree	35.6% (*n* = 36)
Doctoral Degree	37.6% (*n* = 38)
Competency (*n* = 101)	
Competency to treat those with sexual interest in children	49.5% (*n* = 50)
No competency to treat those with sexual interest in children	50.5% (*n* = 51)

*Note.* Dashes designate no data for the specific category.

### Measures

#### Background Questions

Clinicians were asked about their demographic and professional background. We assessed competency in the same way as a past study (Stephens et al., 2021), by asking clinicians what client groups they had competency to treat. Clinicians were considered competent to treat those with sexual interest in children if they indicated that they were competent to do so (yes/no variable).

#### Attitudes Towards Persons With Sexual Interest in Children (APSIC) Scale

The APSIC scale is a 21-item scale developed in collaboration with B4U-ACT for the purposes of this study. The APSIC scale is based off the Attitudes Towards Sex Offenders – 21 (ATS-21) scale developed by Hogue and Harper (2019); however, the APSIC was created to assess attitudes toward those with sexual interest in children (see Appendix A in the Supplementary Materials for APSIC and original ATS-21 items). APSIC items were scored on a 7-point Likert scale from *strongly disagree* to *strongly agree*. Total scores could range from 0 to 126, with higher scores indicative of greater stigmatizing attitudes (*M* = 48.56, *SD* = 15.24). The APSIC demonstrated very good internal consistency (α = .87).

#### Vignettes

Clinicians were randomly assigned to one of four vignettes that provided a description of a hypothetical client with sexual interest in children (see Appendix A in the Supplementary Materials for vignettes). All vignettes portrayed an individual with sexual interest in children who differed in two ways: why the client sought treatment (low mood unrelated to sexual interest or management of sexual interest) and offence history (no offence history or CSA perpetration).

After reading the assigned vignette, clinicians were asked whether they would accept the referral (yes/no), which was the main outcome of interest (willingness to provide treatment). Clinicians who indicated “no” were asked the reason for their decision, to which four options were available: personal beliefs toward individuals with sexual interest in children; lack of competency in treating individuals with sexual interest in children; both; and other.

### Procedure

Clinicians were recruited through Twitter and professional listservs for mental health professionals in North America. To obtain a diverse sample of clinicians, the first author sent an invitation to distribute the survey to various organizations and institutions of different professional backgrounds and competencies. Of those contacted, three organizations agreed to post the survey on their websites. Additionally, clinicians were recruited through snowball sampling, as it was sent to colleagues of the authors who were also asked to distribute it.

Although a power analysis suggested a sample size of 164, recruitment was stopped after 132 clinicians were recruited over five months. We stopped recruitment because we had exhausted potential recruitment venues and despite our best efforts, only one additional clinician participated in the final month the survey was posted, indicating that obtaining a substantial number of additional responses would be unlikely. Further, the data needed to be analyzed for the first author’s master’s thesis (see Roche, 2020).

The survey was hosted on Qualtrics. After providing consent to participate and completing demographics and questions about their background (e.g., competency), clinicians were randomly assigned to one of four vignettes and asked questions about the vignette. Clinicians were also asked about attending a workshop to learn more about sexual interest in children and what they thought would be beneficial to include in such a workshop; however, these responses are not included in the present study since they are not the focus of the research question^1^1Qualitative analyses about the focus of the workshop will be included in a separate paper alongside corresponding responses from those with sexual interest in children, which will highlight the needs of both groups.. Lastly, clinicians completed the APSIC scale.

### Data Analysis

We conducted a series of univariate analyses (chi squares and *t*-tests) to examine whether stigma differed by competency and the independent effects of our variables of interest on willingness to treat. We also conducted a hierarchical binary logistic regression, controlling for client presenting issue by entering it in step one and APSIC score in step two. Effect sizes are provided throughout the results

## Results

### Competency to Treat and Stigma Scores

In accordance with our first hypothesis, those with competency had significantly lower APSIC scores (*M* = 42.24, *SD* = 11.61) than those without competency (*M* = 54.88, *SD* = 15.90), *t*(98) = 4.54, *p* < .001, and the effect was large, *d* = 0.91, 95% CI [0.45, 1.32].

Overall, 77.2% (*n* = 78) of clinicians said they would provide treatment to the client in their vignette. A randomization check showed that clinician competency and stigma score did not significantly differ between vignette groups. Table 2 shows data on willingness to treat and reason for declining referrals based on vignette condition.

**Table 2:** Clinician Vignette Condition and Referral Acceptance

Vignette Condition	Willingness to Treat		Reason for Declining Referral			
	Yes	No	Personal beliefs	Lack of Competency	Both	Other^a^
Vignette 1 (*n* = 23) (general mental health concerns; no offence)	91.3% (*n* = 21)	8.7% (*n* = 2)	–	50% (*n* = 1)	–	50% (*n* = 1)
Vignette 2 (*n* = 26) (general mental health concerns; previous sexual offence)	84.6% (*n* = 22)	15.4% (*n* = 4)	25% (*n* = 1)	50% (*n* = 2)	–	25% (*n* = 1)
Vignette 3 (*n* = 25) (managing sexual interest; no offence)	84% (*n* = 21)	16% (*n* = 4)	–	75% (*n* = 3)	25% (*n* = 1)	–
Vignette 4 (*n* = 27) (managing sexual interest; previous sexual offence)	51.9% (*n* = 14)	48.1% (*n* = 13)	–	61.5% (*n* = 8)	23.1% (*n* = 3)	15.4% (*n* = 2)

*Note.* Dashes designate no data for the specific category. Willingness to treat is in reference to whether clinicians indicated they would accept the client described in the vignette. Only participants who indicated that they would not accept the referral were asked reasons for declining.

^a^Most individuals who endorsed the “other” category cited that their clients consisted of children.

We conducted post-hoc supplementary analyses of univariate effects of competency, stigma, and presenting issue on willingness to treat. Results suggested that those who were unwilling to treat the client had higher stigma scores (*M* = 57.04, *SD* = 16.13) than those willing to treat the hypothetical client (*M* = 46.03, *SD* = 14.10), *t*(98) = 3.18, *p* < .01, and the effect was moderate, *d* = .76, 95% CI [4.14, 17.89]. They were also more likely than expected to not have competency to treat people with sexual interest in children and to have been assigned to the vignette condition where the client wanted help managing their sexual interest and had an offence history (see Table 3).

**Table 3:** Chi-Square Results for Competency and Presenting Issue on Willingness to Treat (N = 101)

Variable	Willingness to Treat	
	No	Yes
Competency^a^	χ^2^(1) = 24.30***	
No	43.1% (*n* = 22; 3.0)^b^	56.9% (*n* = 29; -1.7)
Yes	2.0% (*n* = 1; -3.1)	98.0% (*n* = 49; 1.7)
Vignette Condition	χ^2^(3) = 13.94**	
General mental health concerns; no CSA	8.7% (*n* = 2; -1.4)	93.3% (*n* = 21; 0.8)
General mental health concerns; CSA	15.4% (*n* = 4; -0.8)	84.6% (*n* = 22; 0.4)
Managing sexual interest; no CSA	16.0% (*n* = 4; -0.7)	84.0% (*n* = 21; 0.4)
Managing sexual interest; CSA	48.1% (*n* = 13; 2.8)	51.9% (*n* = 14; -1.5)

*Note.* Chi square analyses were conducted to examine willingness to treat by competency and vignette condition.

^a^Competency was measured by asking clinicians whether they had competency (i.e., experience) in treating those with sexual interest in children. ^b^Standardized residuals are presented in brackets next to cell count.

*p < .05. **p < .01. ***p < .001.

### Correlates of Willingness to Treat

A hierarchical binary regression with willingness to treat as the outcome suggested that relative to the baseline condition (client seeking treatment for managing sexual interest in children with history of CSA perpetration), clinicians were more likely to treat a client from the other three vignette conditions (18.1% of the variance explained in Step 1). For example, those presented with Vignette 1 (general mental health concern, no offence) had nine times the odds of accepting a referral compared with the reference group. Step 2 explained 31.9% of the variance and showed that when controlling for vignette condition, lower stigma scores were associated with increased willingness to treat (see Table 4).

**Table 4:** Results of Hierarchical Binary Logistic Regression Analysis for Vignette Condition and APSIC Scores Predicting Willingness to Treat (N = 101)

Variable	*B*	*SE*	Wald	Odds Ratio	95% CI	χ^2^
Step 1						12.75**
Constant	0.07	0.39	0.04	1.08	–	
Vignette 1	2.23	0.84	7.11**	9.29	[1.81, 47.77]	
Vignette 2	1.63	0.67	5.99*	5.11	[1.38, 18.85]	
Vignette 3	1.58	0.67	5.63*	4.88	[1.32, 18.05]	
Step 2						23.63***
Constant	2.98	1.02	8.53**	19.77	–	
Vignette 1	2.82	0.94	8.94**	16.74	[2.64, 106.20]	
Vignette 2	1.46	0.70	4.34*	4.32	[1.09, 17.15]	
Vignette 3	1.87	0.75	6.20*	6.46	[1.49, 28.06]	
Stigma	-0.06	0.02	9.33**	0.94	[0.91, 0.98]	

*Note.* Vignette 1 = general mental health concern, no offence; Vignette 2 = general mental health, previous sexual offence; Vignette 3 = managing sexual interest in children, no offence; The reference category for the analyses was the vignette where the client wanted treatment to focus on managing sexual interest in children and had committed CSA.

*p < .05. **p < .01. ***p < .001.

## Discussion

The study results were somewhat promising in that 77% of clinicians indicated that they would be willing to treat a client with sexual interest in children. Nonetheless, results suggested that stigma, client presenting issue, and competency were all associated with willingness to treat clients with sexual interest in children. Presenting issue played a role in decision to treat as clinicians were more likely to treat clients in all other vignette conditions, relative to the condition where clients wanted treatment for their sexual interest and had perpetrated CSA. Almost two-thirds who indicated that they would not accept the client stated that it was due to lack of competency and analyses suggested that competency was associated with willingness to treat the client. Although we do not want to imply that clinicians should work outside their area of competency, it is a problem that many clinicians are not receiving education about clients with sexuality-based concerns. According to Reissing and Giulio (2010) sexuality concerns are a cross-cutting competency meaning all clinicians should be able to address them in their clinical work.

It is evident that stigma also played a role in clinical decision making on whether to accept the client even when controlling for presenting issue. Despite this, there is unaccounted variance in our model and additional factors might help better predict willingness to treat. For instance, uncertainty around mandatory reporting may be a factor in why clinicians are hesitant to provide treatment (Stephens et al., 2021). Although reporting legislation in North America is similar across provinces and states, ambiguity of legislation may cause confusion and decrease clinician willingness to treat populations who may be “at risk” to commit an offence, such as those with sexual interest in children (McPhail et al., 2018).

The findings highlight the importance of stigma in clinician-decision making for clients with sexual interest in children. The results are consistent with Stephens et al. (2021) who found that clinicians with higher stigma toward those with sexual interest in children were more likely to file a mandatory report when individuals disclosed their sexual interest in children. Clinicians are not immune to stigma, which is an important target when considering how to increase willingness of clinicians to treat this group. The importance of stigma is further supported by findings that clinician stigma is identified as a barrier to help-seeking among people with sexual interest in children (Levenson & Grady, 2019) and lack of help-seeking has been suggested to increase risk of CSA (Jahnke, Schmidt, et al., 2015).

One implication of this study is the need for clinician training. To increase access to mental health services for this underserved group, it would be important to curate a stigma-targeting workshop to increase clinician competency and willingness to work with those with atypical sexual interests, including sexual interest in children. A workshop should integrate information on those with sexual interest in children and their treatment needs and include elements of social contact to facilitate stigma reduction (Corrigan et al., 2012). The need for social contact is further reinforced by our finding that clinicians with competency to treat those with sexual interest in children (and thereby had greater exposure to this group) had lower stigma scores than those without competency. Social contact can be implemented by having those with sexual interest in children attend the workshop, for example, as a speaker in a panel. Privacy concerns could be addressed by individuals attending via conference call without video. Certain workshops, such as one run by the Association for Sexual Abuse Prevention (ASAP), have already integrated social contact in this way.

### Limitations and Future Directions

A limitation was the use of self-report data and convenience sampling, which resulted in half of the sample having competency to treat those with sexual interest in children. Our sample size was also limited, the impact of which was demonstrated by the presence of wide confidence intervals when examining the impact of vignette condition on willingness to treat, suggesting unstable effects. While the first author attempted to recruit from various organizations and institutions of different professional backgrounds, most invitations were not responded to. A sample with less experience working with people with sexual interest in children may result in different outcomes (e.g., stigma scores may have been higher if more generalist clinicians were included). Overall, the generalizability of our results is limited, and the results should be viewed as preliminary. Future research should seek to replicate these analyses with a larger, more diverse sample by recruiting for a longer period and trying to recruit a more representative sample.

Although there are strengths of the APSIC (e.g., input from B4U-ACT, modified from an existing scale), it is important to highlight that the APSIC has not undergone rigorous testing of its psychometric properties. Future research is required before the APSIC can be established as a valid measure of clinician stigma. We also presented the vignettes prior to the APSIC scale, which could have primed clinician’s responses to the stigma scale. In the future, researchers should randomize the order so that half of the sample are presented with the vignettes first, and the other half second.

## Supplementary Materials

The Supplementary Materials contain the APSIC-21 items and vignettes presented to clinician participants in Roche and Stephens (2021) (for access see Index of Supplementary Materials below).



RocheK.
StephensS.
 (2021). Supplementary materials to "Clinician stigma and willingness to treat those with sexual interest in children"
[Additional information]. PsychOpen. 10.23668/psycharchives.5369
PMC1178943439902157

## Data Availability

Data is unavailable in order to uphold the informed consent statements that data would not be shared with anyone except the research team, and that raw data would not be published.

## Author Note

This study was completed as part of the requirements for the first author’s master’s thesis. The full thesis is composed of two studies and can be found in Roche (2020).
